# Radiomics-Driven CBCT Texture Analysis as a Novel Biosensor for Quantifying Periapical Bone Healing: A Comparative Study of Intracanal Medications

**DOI:** 10.3390/bios15020098

**Published:** 2025-02-09

**Authors:** Diana Lorena Garcia Lopes, Sérgio Lúcio Pereira de Castro Lopes, Daniela Maria de Toledo Ungaro, Ana Paula Martins Gomes, Nicole Berton de Moura, Bianca Costa Gonçalves, Andre Luiz Ferreira Costa

**Affiliations:** 1 Postgraduate Program in Dentistry, Dentomaxillofacial Radiology and Imaging Laboratory, Department of Dentistry, Cruzeiro do Sul University (UNICSUL), São Paulo 01506-000, SP, Brazil; dilomagar@hotmail.com; 2 Department of Diagnosis and Surgery, São José dos Campos School of Dentistry, São Paulo State University (UNESP), São José dos Campos 12245-000, SP, Brazil; sergioluciolopes@gmail.com (S.L.P.d.C.L.); nicole.berton@unesp.br (N.B.d.M.); biaunespsjc@gmail.com (B.C.G.); 3 Department of Restorative Dentistry, Institute of Science and Technology, São Paulo State University (UNESP), São José dos Campos 12245-000, SP, Brazil; danielamt_ungaro@yahoo.com.br (D.M.d.T.U.); paula.gomes@unesp.br (A.P.M.G.)

**Keywords:** biomarkers, diagnostic imaging, image processing, periapical periodontitis, radiomics

## Abstract

This study aimed to evaluate the effectiveness of two intracanal medications in promoting periapical bone healing following endodontic treatment using radiomics-enabled texture analysis of cone-beam computed tomography (CBCT) images as a novel biosensing technique. By quantifying tissue changes through advanced image analysis, this approach seeks to enhance the monitoring and assessment of endodontic treatment outcomes. Thirty-four single-rooted teeth with pulp necrosis and periapical lesions were allocated to two groups (17 each): calcium hydroxide +2% chlorhexidine gel (CHX) and Ultracal XS^®^. CBCT scans were obtained immediately after treatment and three months later. Texture analysis performed using MaZda software extracted 11 parameters based on the gray level co-occurrence matrix (GLCM) across two inter-pixel distances and four directions. Statistical analysis revealed significant differences between medications for S [0,1] inverse difference moment (*p* = 0.043), S [0,2] difference of variance (*p* = 0.014), and S [0,2] difference of entropy (*p* = 0.004). CHX treatment resulted in a more organized bone tissue structure post-treatment, evidenced by reduced entropy and variance parameters, while Ultracal exhibited less homogeneity, indicative of fibrous or immature tissue formation. These findings demonstrate the superior efficacy of CHX in promoting bone healing and underscore the potential of texture analysis as a powerful tool for assessing CBCT images in endodontic research.

## 1. Introduction

In the field of endodontics, the primary objective is to remove or significantly diminish the microbiota within the root canals by means of biomechanical preparation in order to foster the healing of periradicular tissues [1]. The significance of this objective stems from the established understanding that microorganisms and their metabolic by-products are predominantly responsible for pulpal alterations and the development of periapical lesions, which can lead to pulp necrosis and infection [2]. Specifically, the prevalence of Gram-negative anaerobic microorganisms has been correlated with root canals, which exhibit radiographically visible periapical lesions, a situation exacerbated by the release of endotoxins during death or multiplication of bacteria [3]. These endotoxins stimulate a series of inflammatory responses, thus contributing to bone resorption associated with periapical lesions [2,3].

Among the advancements in endodontic treatments, various irrigation solutions and intracanal medications have been used to combat persistent infection within the root canal system [4]. Sodium hypochlorite stands out for its broad-spectrum antimicrobial activity and ability to dissolve necrotic tissue, making it a widely used irrigation solution [5]. Additionally, calcium hydroxide has been recognized for its antimicrobial efficacy and its role in promoting tissue repair, in which one can highlight the multifaceted approach to eliminating root canal pathogens [4,5]. Despite these therapeutic advancements, the challenge of completely eradicating microorganisms and endotoxins from the root canal system remains, which emphasizes the importance of effective post-operative interventions. Conventional diagnostic methods, particularly periapical radiography, have been instrumental in the diagnosis, planning and evaluation of endodontic treatments. However, the limitations of two-dimensional imaging in accurately detecting and measuring periapical lesions have necessitated the exploration of more sophisticated diagnostic tools [6]. Cone beam computed tomography (CBCT) has emerged as a superior alternative to conventional ones, offering detailed three-dimensional images without overlap of anatomical structures associated with two-dimensional imaging [7]. This study introduces texture analysis as a novel approach to assess bone repair in teeth with periapical lesions following endodontic treatment.

Texture analysis, a computer-assisted technique, shows the patterns and distributions of pixel intensities within digital images to quantitatively assess structural changes [8,9]. By using texture analysis, this study aims to evaluate the effectiveness of two different types of intracanal medication in the healing process. This innovative methodology not only enhances the understanding of bone healing dynamics but also contributes to optimizing endodontic treatment outcomes, thus underscoring the potential of texture analysis in promoting advances in endodontic research and practice.

This study aimed to evaluate the effectiveness of two intracanal medications in promoting periapical bone healing following endodontic treatment using radiomics-enabled texture analysis of CBCT images as a novel biosensing technique. This approach seeks to quantitatively assess and differentiate tissue changes, potentially enhancing the monitoring of endodontic treatment outcomes.

## 2. Materials and Methods

This study was approved by the Ethics Committee of the São Paulo State University (UNESP) according to protocol number 50377321.1.0000.0077 and conducted in accordance with the ethical principles for medical research involving human subjects as described in the Declaration of Helsinki. Prior to inclusion in this study, written informed consent was obtained from all subjects.

This retrospective study used a sample of images from a previous research project. The original study focused on determining the estimated time for bone repair in endodontically treated teeth with periapical lesions and comparing the volume of periapical lesions after the use of two types of intracanal medication.

### 2.1. Patient and Tooth Selection for Endodontic Treatment

Thirty-four single-rooted teeth with pulp necrosis and periapical lesions were selected from patients referred to the Endodontics Clinic of the Department of Restorative Dentistry at the Institute of Science and Technology of São José dos Campos, UNESP, for endodontic treatment. The selection was based on pre-established inclusion and exclusion criteria. Before treatment, we evaluated and recorded the condition of each tooth’s crown and its occlusal state.

Exclusion criteria encompassed patients who had used antifungals and/or antibiotics (up to 3 months before the study), who were unavailable for radiographic and tomographic follow-up 3 months after treatment completion or who had teeth with periodontal disease or root fractures. Patients with a history of systemic diseases that could affect bone metabolism, such as osteoporosis or diabetes mellitus, were excluded from the study. We also excluded patients who were pregnant, had a history of bisphosphonate use, or were undergoing radiation therapy in the head and neck region. Teeth that experienced significant changes in crown condition or occlusal state during the study period were excluded from the final analysis to minimize potential confounding factors.

### 2.2. CBCT Image Acquisition

CBCT scans were acquired by using an i-CAT Next Generation scanner (Imaging Sciences International, Hatfield, PA, USA) at the Radiology Clinic of the Institute of Science and Technology of the UNESP. The scanning protocol used was the following: field of view (FOV) of 6.0 cm × 16.0 cm encompassing the dental arch of interest and voxel size of 0.25 mm. The average acquisition time was 14.0 s.

To study the variation in bone formation in the periapical region, all the patients underwent CBCT examinations at two different treatment stages:

T1: immediately after the completion of endodontic treatment.
T2: three months after treatment completion.

### 2.3. Intracanal Medication for 14 Days

After biomechanical preparation, the teeth were divided into two groups (n = 17) depending on the intracanal medication (ICM) used, as follows:

Group 1: calcium hydroxide (Biodinâmica, Ibipurã, PR, Brazil) + 2% chlorhexidine gel (Concepts V—Ultradent Products, South Jordan, UT, USA).
Group 2: Ultracal XS^®^ (Ultradent Products, Inc.).

In Group 1, the medication combination (calcium hydroxide + 2% chlorhexidine gel) was prepared in a 1:1 volume ratio (toothpaste consistency) and delivered into the root canal by using files and Lentulo spirals (Dentsply/Maillefer Instruments SA, Ballaigues, Switzerland) until complete root canal filling. The teeth were then sealed with a layer of pure calcium hydroxide, followed by a layer of Coltosol (Vigodent, Rio de Janeiro, RJ, Brazil) and a temporary restoration by using glass ionomer cement (Vidrion R—S.S. White Artigos Dentários Ltd., Rio de Janeiro, RJ, Brazil).

In Group 2, Ultracal XS^®^ was inserted by using NaviTip from the kit, with additional insertion by means of manual files and Lentulo spirals to ensure complete filling.

The assignment of medications to each tooth was performed using a simple alternating method. As patients were enrolled in the study, their teeth were alternately assigned to either Group 1 or Group 2. This method ensured an equal distribution of teeth between the two groups while maintaining a straightforward and easily replicable randomization process. This method ensured an equal distribution of teeth between the two groups while maintaining a straightforward and easily replicable randomization process. The intracanal medication was maintained for a period of 14 days in both groups.

### 2.4. Texture Analysis

CBCT images in DICOM (Digital Imaging and Communications in Medicine) format were extracted from the database and imported into a high-performance notebook computer (MacBook Pro (Cupertino, CA, USA), Intel^®^ CoreTM i5, 2.4 GHz, 4 GB, 1067 MHz, DDR3 processor) running Microsoft Windows. An evaluator with ten years of experience in CBCT interpretation reviewed the images and selected central sections that clearly displayed the lesions. For each tooth, three image slices were chosen: the central slice of the lesion and two adjacent slices (one in the lateromedial direction and one in the superoinferior direction). This approach was adopted to ensure consistent volumetric representation of the lesion for texture analysis, balancing lesion representativeness with computational efficiency. These slices were processed and converted to BMP (bitmap) format by using OnDemand3D software, version 1.0 (CyberMed Inc., Seoul, South Korea) [10].

**Figure 1:**
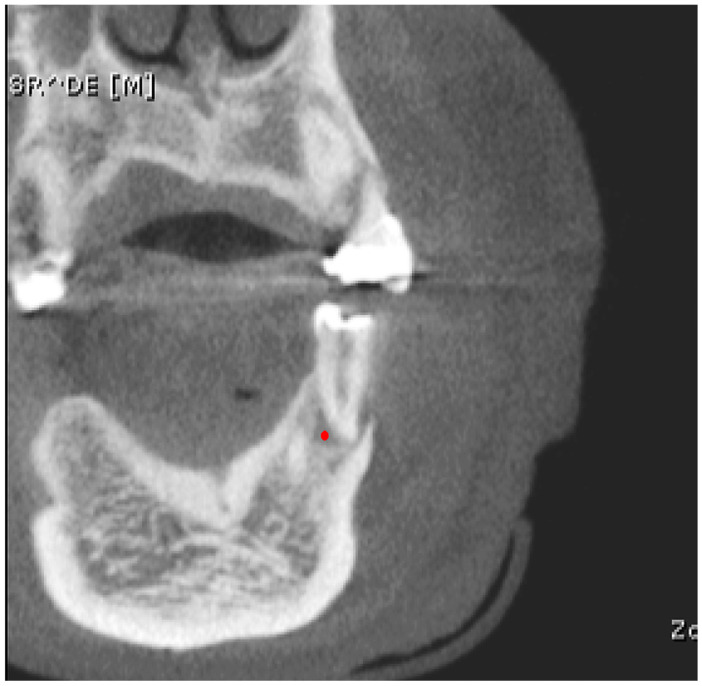
CBCT slice demonstrating the placement of the circular region of interest (ROI) for texture analysis of periapical lesions using MaZda software. The red dot indicates the center of the periapical lesion, determined at the intersection of the lateromedial and superoinferior lines. A circular ROI with a diameter of 44 pixels was centered on this point to ensure only lesion tissue was included in the analysis.

Next, a second evaluator well-versed in CBCT image analysis who was unaware of clinical and diagnostic information conducted the texture analysis. The BMP format images were imported into MaZda software (version 4.6) for texture feature calculation. A circular region of interest (ROI) of 44 pixels in diameter was manually delineated at the center of the lesion (point marked in red) on the frontal image to ensure that only the lesion tissue was included (Figure 1). The center of the periapical lesion was determined at the intersection of the lateromedial and superoinferior lines.

This study used a gray-level co-occurrence matrix (GLCM), which is a square matrix with dimensions equal to the image’s gray levels, to reveal the spatial distribution of gray levels in the image texture [11]. This mathematical approach, based on Haralick’s method, calculates parameters according to the frequency of specific pairs of pixel values in the image [10].

Eleven texture parameters were extracted as follows: angular second moment (AngScMom), contrast, correlation (Correlat), difference of entropy (DifEntrp), difference of variance (DifVarnc), entropy, inverse difference moment (InvDfMom), sum of average (SumAverg), sum of entropy (SumEntrp), sum of squares (SumOfSqs), and sum of variance (SumVarnc). These were calculated for two inter-pixel distances (d1 = 1, d2 = 2) and four image directions (horizontal, vertical, 45°, and 135°). The distances were arranged in four directions, resulting in the following positions: S(1,0), S(0,1); S(2,0), S(0,2); and S(3,0), S(0,3) [10]. The selection of these specific texture parameters and analysis methods was based on their ability to provide a comprehensive characterization of bone tissue texture in CBCT images. This approach, as demonstrated by Gonçalves et al. [10], allows for a thorough examination of the spatial relationships and distribution of gray levels within the region of interest. The use of multiple inter-pixel distances and directions enables the capture of texture information at different spatial scales and orientations, which is fundamental for analyzing the complex patterns of bone microarchitecture. The GLCM method, from which these parameters are derived, is particularly effective in quantifying subtle changes in bone structure that may not be apparent through conventional image analysis. By employing this multifaceted texture analysis approach, this study aimed to detect and quantify changes in bone texture associated with the healing process following different endodontic treatments, potentially improving our ability to assess treatment outcomes and periapical bone regeneration.

**Figure 2:**
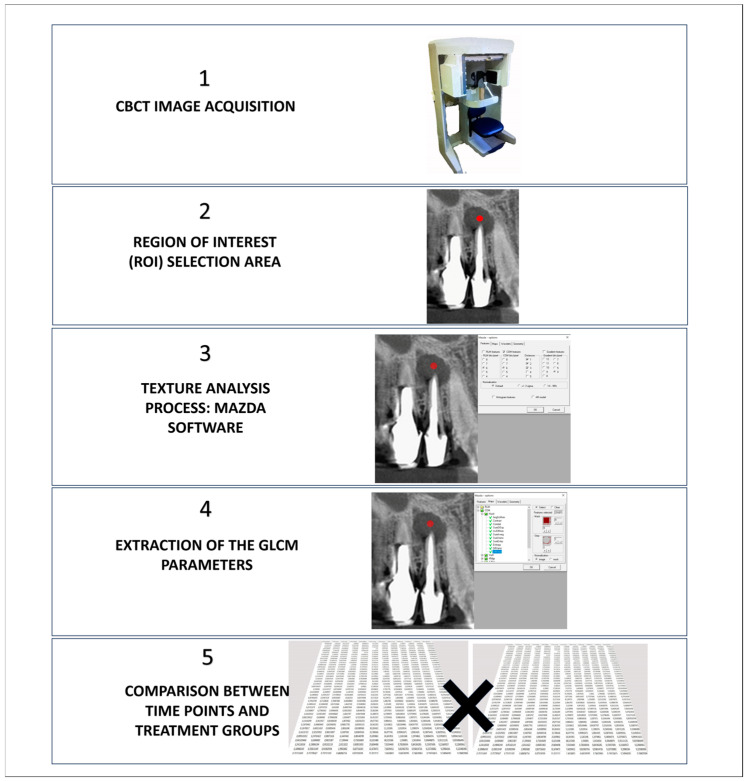
Schematic representation of the texture analysis workflow for quantifying periapical bone healing. (1) The process begins with CBCT image acquisition at two time points (T1: immediately after treatment, T2: 3 months post-treatment), (2) followed by region of interest (ROI) selection. (3) Texture analysis is performed using MaZda software, (4) extracting 11 GLCM parameters. (5) Statistical analysis compares these parameters between the two medication groups and time points.

To illustrate the complete workflow of the texture analysis process and quantification of periapical bone healing, we developed a schematic diagram (Figure 2). This diagram summarizes the main steps of our method, from CBCT image acquisition to the final interpretation of results, highlighting the key parameters analyzed and how they relate to the assessment of bone healing.

### 2.5. Statistical Analysis

An exploratory data analysis was conducted by using summary measures (mean and standard deviation) and graphical representations. Time points and medications were compared for each parameter by using ANOVA with rank transformation, as the parameters did not exhibit normal distribution. The significance level for all analyses was set at 5%. Statistical analyses were performed by using R Core Team (2023) software (R: A Language and Environment for Statistical Computing. R Foundation for Statistical Computing, Vienna, Austria).

## 3. Results

This study included 34 patients of both sexes, of which 80% were female. Thirteen patients were treated with CHX, whereas 11 were treated with Ultracal medication. In Group 1, eight patients (62%) were female, and in Group 2, all were female.

Given that no high correlation was observed between the directions of the same parameter, all directions were analyzed separately.

**Figure 3:**
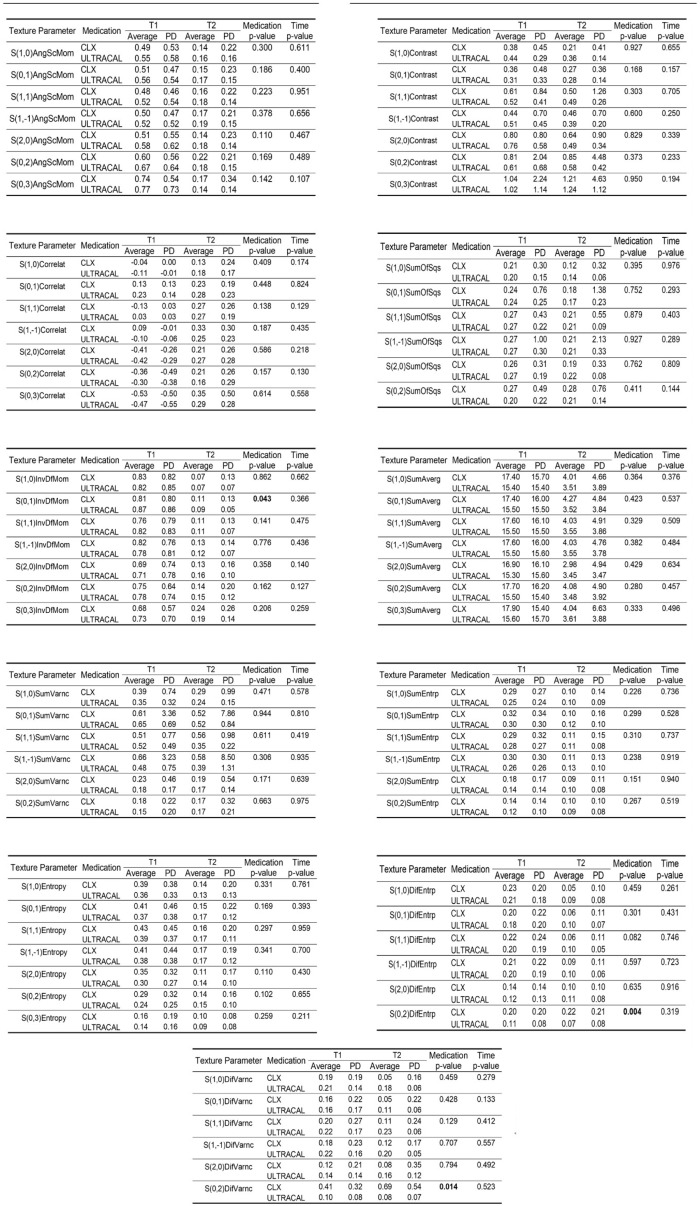
Descriptive measures and comparative analysis of 11 texture parameters for two intracanal medications (CHX and Ultracal) across two time points (T1 and T2). Parameters include angular second moment (AngScMom), contrast, correlation (Correlat), difference of entropy (DifEntrp), difference of variance (DifVarnc), entropy, inverse difference moment (InvDfMom), sum of average (SumAverg), sum of entropy (SumEntrp), sum of squares (SumOfSqs), and sum of variance (SumVarnc). Values represent means and standard deviations (SDs) for each parameter, along with *p*-values for medication and time comparisons.

**Figure 4:**
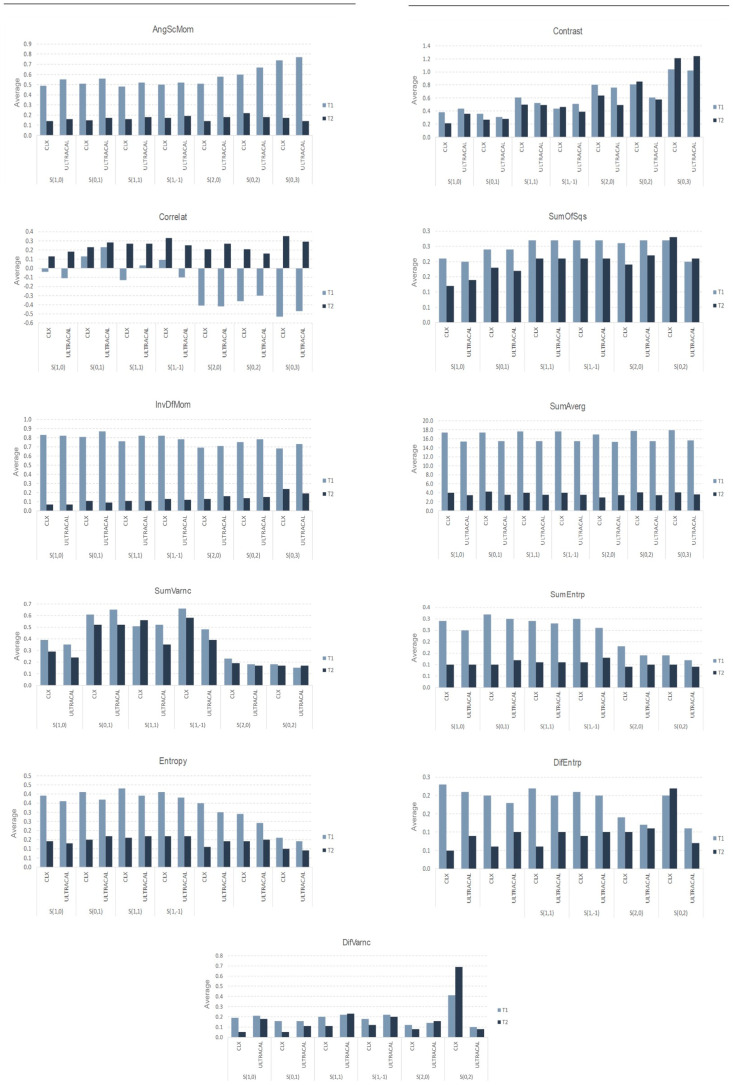
Graphical representations of changes in 11 texture parameters between two time points (T1 and T2) for two intracanal medications (CHX and Ultracal). Parameters shown are angular second moment (AngScMom), contrast, correlation (Correlat), sum of squares (SumOfSqs), inverse difference moment (InvDfMom), sum of average (SumAverg), sum of variance (SumVarnc), sum of entropy (SumEntrp), entropy, difference of entropy (DifEntrp), and difference of variance (DifVarnc). Bars represent mean values at each time point for both medications.

Figure 3 and Figure 4 show the descriptive measures and comparison between medications and time points for the 11 parameters as follows: angular second moment, contrast, correlation, difference of entropy, difference of variance, entropy, inverse difference moment, sum of average, sum of entropy, sum of squares, and sum of variance.

Statistically significant differences were observed between the medications for the following parameters:

- S(0,1) InvDfMom (*p*-value = 0.043): Ultracal medication showed a greater post-treatment reduction compared with CHX.
- S(0,2) DifVarnc (*p*-value = 0.014): Ultracal medication showed a post-treatment reduction, whereas CHX showed an increase.
- S(0,2) DifEntrp (*p*-value = 0.004): Ultracal medication showed a post-treatment reduction, whereas CHX showed an increase.

## 4. Discussion

The capacity to analyze the dimensional reduction of inflammatory periapical lesions through endodontic treatment follow-up, whether by two-dimensional images (i.e., periapical radiographs) or three-dimensional examinations (i.e., cone-beam computed tomography), has been previously studied by using linear or volumetric measurements, respectively [12,13,14,15].

Changes in the internal imaging features of alveolar bone related to inflammatory periapical lesions reflect either a reduction or an increase in bone structure or a combination of both. A quantitative reduction in bone structure is observed as an increase in radiolucency due to a decrease in the number and density of the existing trabeculae. Conversely, bone augmentation is seen as an increased radiopacity (i.e., sclerosis) resulting primarily from an increase in thickness, density and number of trabeculae [16].

Nevertheless, there is a gap in the literature regarding the qualitative evaluation of the bone healing process of inflammatory periapical lesions in relation to the medicinal approach to be used in endodontic treatment. In this context, the present study presents a novel methodology in which an investigation of inflammatory periapical lesions was conducted according to the type of intracanal medication and after 3 months of endodontic treatment. This was achieved through texture analysis of CBCT images of these lesions, a method allowing for comparison between the effects of two ICMs, namely, CHX and Ultracal.

The choice of CBCT images for this study was based on their ability to provide a more reliable assessment without structure overlapping, which could mask the real content of periapical lesions, as occurs in conventional radiographs [12,17].

The results indicated that among the 11 texture parameters analyzed by using GLCM for periapical bone lesions, 3 of them [i.e., InvDfMom, DifEntrp, and DifVarnc] showed statistically significant differences between the two study groups, considering the time elapsed since the completion of endodontic treatment. A similar behavior was observed in these parameters, as their values for bone were decreased in the regions of periapical lesions in Group 2 (Ultracal) compared with Group 1 (CHX). In other words, patients who were treated with Ultracal medication in their endodontic treatment had periapical bone regions with previous inflammatory lesions, as qualitative bone aspects were differentiated by these texture parameters in the group treated with CHX medication.

One must consider the meaning [18] of each of these three parameters to understand these results from the correlation between them and the properties of the studied medications. This may result in differences, thus enabling quantitative evaluation of the bone. It should be remembered that the texture analysis technique allows this type of analysis to be performed, as it is an analytical method based on markers [parameters] and behavior of pixels within a determined ROI in the images by comparing the values of each marker at different distances correlated to pixels located in the central sites of the ROI [19,20].

Image analysis of the bone corresponding to periapical lesion areas of teeth treated with Ultracal medication revealed, after three months of post-endodontic treatment, a lower value of InvDfMom compared with that in Group 1. This parameter governs the degree of homogeneity in the distribution of image gray levels, as its values decreased in the lesion regions of Ultracal-treated teeth compared with those treated with CHX. Therefore, it can be inferred that the bone in the region showed heterogeneous patterns compared to the newly-formed bone in the periapical regions of teeth treated with calcium hydroxide + 2% chlorhexidine gel [Group 1]. This suggests that bone trabeculae and medullary spaces failed to become organized so that conclusive aspects of a more standardized scar tissue could potentially indicate tissue with increased medullary spaces, fewer bone trabeculae or even fibrous tissue [fibrous scar].

These findings are reinforced by Costa et al. [9], who analyzed dental implant stability by evaluating texture patterns of the bone site at the implant placement bed. In their study, InvDfMom was a texture marker showing reduced values in sites where implant torque values were lower, suggesting less dense bone for implant osseointegration and indicating components similar to those of immature bone tissue or even fibrous tissue.

Analogous to InvDfMom, this study showed reduced values of DifEntrp for periapical bone lesions in Group 2. This parameter indicates differences in the disorganization of gray level distribution, in which a difference in entropy [rather than pure entropy] indicates that the greater the disorder, the lower its value. Our results corroborate those observed by De Rosa et al. [11], who investigated the potential of texture analysis techniques to differentiate between apical radicular cysts and periapical granulomas. They found that various texture parameters, including DifEntrp, could be used for such differentiation in CBCT images as their lower values were statistically significant for granuloma lesions, thus indicating a higher likelihood of fibrous tissue exhibiting this behavior.

Similar findings were observed in studies by Queiroz et al. [21] and Gonçalves et al. [10], who evaluated CBCT images to assess the ability of texture markers to identify affected versus healthy tissues in bone regions bordering medication-related osteonecrosis and furcation lesions, respectively. Their findings confirmed this technique’s potential as a promising means of qualitative image evaluation besides allowing pure optical analysis. In both studies, texture parameters [including lower values of DifEntrp and InvDfMom] indicated that the tissue was sufficiently altered compared with the unaffected bone for the same parameters in the characterization of inflammatory tissue, more closely resembling a contaminated bone. In our study, this can be interpreted as a reduced ability of Ultracal to promote pure bone repair of inflammatory periapical lesions.

DifVarnc is a texture parameter, like those previously described, showing statistically significant differences between the groups by characterizing the dispersion of gray level differences in the image. Higher values indicate greater dispersion of gray levels, whereas lower values indicate the opposite. Considering bone analysis, high dispersion of gray levels in the segmented region indicates a balanced presence of trabeculae and medullary spaces, characterizing a more organized and vascularized bone tissue with histological aspects corresponding to type II bone. Conversely, lower values of DifVarnc [i.e., less dispersion of gray levels] characterize a bone pattern compatible with type IV bone in osteogenic terms, which is quite spongy, with fewer trabeculae [22,23,24] and predominantly lower density regions, potentially representing less mineralized bone or even fibrous tissue.

The previously detailed results indicate that the texture analysis technique used in CBCT images allowed a new qualitative approach to assess the effects of two intracanal medications [i.e., CHX and Ultracal] on the bone neoformation process of periapical lesions. This approach signals that in lesions associated with Ultracal medication, the resulting bone aspect showed less homogeneity and greater structural disorganization three months after endodontic treatment, including an aspect resembling more closely a tissue less likely to match that of bone tissue but rather fibrous tissue or immature bone.

The two types of ICMs addressed in this study were chosen due to their abilities relative to calcium hydroxide in neutralizing endotoxins by elevating the pH of the medium [i.e., alkalinizing action], which alters the cytoplasmic membrane of bacteria [i.e., antibacterial action] due to high pH, and by stimulating mineralization through alkaline phosphatase [i.e., mineralization induction] [25]. However, it should be emphasized that despite the presence of calcium hydroxide in both groups, CHX-treated teeth included its association with 2% chlorhexidine gel, whereas Ultracal-treated teeth had the presence of 35% calcium hydroxide in aqueous solution. This fact may precisely be an indicator related to the difference found through texture analysis of the qualitative bone aspects in post-endodontic treatment lesions, as calcium hydroxide associated with chlorhexidine can potentiate antimicrobial effects on Gram-positive and Gram-negative bacteria while preserving its biocompatibility [26,27], which does not occur with calcium hydroxide alone in an aqueous medium.

Although this fact has already been scientifically proven, our study becomes important as it presents a qualitative analytical approach to the effects of these two ICMs on the healing process of the corresponding periapical lesions through an objective method already validated in different imaging modalities, including conventional radiographs, computed tomography and magnetic resonance imaging [9,11,21,28]. Therefore, texture analysis has not been used until now as a tool for endodontic evaluation of periapical lesions, thus demonstrating the novel nature and importance of the present study.

It is important to note that the present study has some limitations. First, the sample size of 34 single-rooted teeth (17 per group), while sufficient to detect significant differences in several texture parameters, may limit the generalizability of our findings. A larger sample size in future studies could potentially reveal more subtle effects and increase the robustness of our conclusions. Additionally, only single-rooted teeth were treated with two different ICMs, not considering factors such as age, gender, stress, nutrition, vitamin intake, quality of coronal sealing, as well as hypertension, osteoporosis, and diabetes mellitus, among others. These factors could additionally influence the periapical healing process [29,30]. Furthermore, all CBCT scans in this study were performed using a single-machine model. It is important to acknowledge that relative shooting values and image characteristics may differ across various CBCT machine brands and models, which could affect the generalizability of our texture analysis results. Future multi-center studies utilizing different CBCT machines could help validate the robustness of our findings across various imaging platforms and further strengthen the applicability of this texture analysis approach in clinical practice.

## 5. Conclusions

This study demonstrates the potential of radiomics-enabled texture analysis of CBCT images as a novel biosensing technique for quantitative assessment of periapical bone healing. Our findings indicate that this method can effectively differentiate between tissue characteristics resulting from different intracanal medications. Specifically, teeth treated with CHX exhibited more uniform features consistent with organized bone tissue, while those treated with Ultracal showed less homogeneity, suggesting fibrous or immature tissue. This radiomics-based approach not only highlights the efficacy of CHX as an intracanal medication but also showcases the analytical capability of CBCT-based texture analysis as a promising biosensing platform for non-invasive, quantitative evaluation of endodontic treatment outcomes.

## Data Availability

The datasets generated and/or analyzed during the current study are available from the corresponding author upon reasonable request.
